# Moving away from deficiency models: Gradiency in bilingual speech categorization

**DOI:** 10.3389/fpsyg.2022.1033825

**Published:** 2022-11-24

**Authors:** Ethan Kutlu, Samantha Chiu, Bob McMurray

**Affiliations:** ^1^Department of Psychological and Brain Sciences, University of Iowa, Iowa City, IA, United States; ^2^Department of Linguistics, University of Iowa, Iowa City, IA, United States

**Keywords:** speech perception, gradiency, categorical perception, bilingualism, sound acquisition, heritage bilingualism

## Abstract

For much of its history, categorical perception was treated as a foundational theory of speech perception, which suggested that quasi-discrete categorization was a goal of speech perception. This had a profound impact on bilingualism research which adopted similar tasks to use as measures of nativeness or native-like processing, implicitly assuming that any deviation from discreteness was a deficit. This is particularly problematic for listeners like heritage speakers whose language proficiency, both in their heritage language and their majority language, is questioned. However, we now know that in the monolingual listener, speech perception is gradient and listeners use this gradiency to adjust subphonetic details, recover from ambiguity, and aid learning and adaptation. This calls for new theoretical and methodological approaches to bilingualism. We present the Visual Analogue Scaling task which avoids the discrete and binary assumptions of categorical perception and can capture gradiency more precisely than other measures. Our goal is to provide bilingualism researchers new conceptual and empirical tools that can help examine speech categorization in different bilingual communities without the necessity of forcing their speech categorization into discrete units and without assuming a deficit model.

## Introduction

Listeners encounter highly variable speech signals every day. Much of the research on speech perception has focused on understanding this problem of lack of invariance – how does a given listener categorize a highly variable acoustic signal into discrete units like features, phonemes or words to extract the linguistic information relevant for that utterance? For a long time, these issues were investigated under the umbrella of categorical perception (henceforth CP; Liberman et al., 1957). Theoretically, CP argues that perception—the pre-categorical auditory encoding—is warped by the presence of categories. One consequence of this is that during speech perception, listeners discard continuous acoustic information that is irrelevant to category identity and only perceive the category.

For example, voice onset time (VOT) is a continuous cue that distinguishes voiced and voiceless/aspirated consonants across many languages (Lisker and Abramson, 1964; Lisker, 1986; Abramson and Whalen, 2017). It is defined as the period of time between the release of a stop consonant and the onset of voicing. In English, voiced sounds have VOTs near 0 msec, and voiceless near 60 msec, though this varies cross linguistically. Even though VOT scales continuously, CP argued that English-speaking listeners are less capable of hearing the difference between VOTs of 40 and 50 msec (both of which indicate a voiceless sound) than the difference between 15 and 25 msec (which spans the boundary), despite the fact that each contrast has an equivalent physical distance.

CP led to two contributions that shaped subsequent work on multilingualism. The first was methodological: the extensive use of speech continua and forced choice tasks along with a set of theoretical assumptions about how to interpret them. The second was theoretical: CP led to an implicit view that a sort of quasi-discrete representation of speech was desirable and any deviation from that may represent a deficit. Importantly, this representational system emerges during the first year of life. This impact can be seen in two examples.

First, research on bilinguals has long known that adult L2 learners face challenges in acquiring the categories of their second language (Strange and Shafer, 2008). The question is why? Classic developmental work argued that speech categories are formed during the first year of life and that the emergence of these categories and their structure was associated with a sensitive period (Werker and Tees, 1984) (though see McMurray, 2022). If we assume CP as a model of speech perception, this can then explain adult learners: many new L2 distinctions comprise within-category distinctions in the native language (e.g., the English l/r distinction which lies within the Japanese category). If listeners cannot hear these distinctions due to the effect of early experience, this can explain why L2 learning is so hard.

More broadly, the assumption of CP (and the methods) also served as a sort of linking hypothesis to understanding bilingual abilities. In particular, the forced-choice task has been extensively interpreted such that a steeper slope (i.e., categorical) reflects better perceptual encoding, and a shallower slope (i.e., gradient) reflects a deficiency in perceptual encoding. Consequently, even a slight deviance from monolingual-like performance led to discussions of whether bilinguals can form monolingual-like categories. That is the steep slope is considered ideal and any departure reflects a limitation.

However, this sort of simple framing may be inappropriate when we consider the wide variety of forms that bilingualism can take. In a heritage bilingual context, the first years of life might have more emphasis on the heritage language compared to the majority language. Nonetheless, their continued exposure to the majority language may overcome this background. Alternatively, the dynamics between the heritage language and the majority language might change depending on the bilingual context (e.g., code-switching vs. a more linguistically homogenous context). In these cases, it may be more appropriate to evaluate cues like VOT gradiently across different contexts, rather than attempting to impose a single sharp (and inflexible) boundary.

While work on bilingualism has operated on the assumption of CP, research on monolingual adults has begun to move away from it [for a review (McMurray, n.d.)]. As we describe, this work suggests that adult listeners show robust sensitivity to within-category differences, and that speech categories may be highly gradient. In fact, unlike the claims made by categorical perception, this more recent work suggests that having a shallow slope (i.e., being gradient) is not an indicator of deficiency. On the contrary, it might be the marker of better information encoding. It also proposes new methods (and new ways of understanding existing measures) that may allow more sensitive ways to probe individual differences and are more aligned with this theoretical development.

The goal of this manuscript is to challenge the assumption of CP in bilingualism research, particularly in heritage bilingualism. We first describe the debates over CP in monolingual speech perception. We then focus on how assumptions of CP impacted bilingualism research and how it led to a deficiency model of bilingual speech perception. We will then introduce the Visual Analogue Scaling task (VAS task), which has been a trademark of our research group, to examine speech perception in developing children (both monolingual and bilingual) and adults (monolingual, bilingual, and cochlear implant users). We will present preliminary data from an ongoing experiment that exemplifies how the VAS task can capture profiles of gradient speech perception in bilinguals’, and we introduce new statistical modeling that builds the notion of individual variability into the analysis. The ultimate goal of this manuscript is to move bilingualism research away from the theoretical assumptions produced by categorical perception and show how methodological reconsiderations are necessary to fully capture different bilingual profiles without the deficiency model.

## Categorical perception in language science research

Historically, a large majority of speech perception research has focused on the problem of lack of invariance (Kluender, 1994; Liberman and Whalen, 2000; Perkell and Klatt, 2014). This problem arises from the fact that the same phoneme varies in its own acoustic manifestation depending on the speaker’s speech rate, phonetic context, and many other variables. Moreover, the same bundle of acoustic cue values can be consistent across multiple phonemes. The problem then is how can a listener efficiently map a continuous acoustic signal onto a set of discrete units (e.g., phonemes) in the face of a non-invariant mapping between individual cue values and categories?

CP (Liberman et al., 1957) was central to early theoretical approaches to this problem. CP was initially an empirical phenomenon which was observed when listeners showed poor discrimination for two speech sounds that arose from the same category, but good discrimination for tokens that span the boundary, even when the acoustic difference was the same. For instance, VOT is a critical cue for stop consonant voicing. Voiced sounds like /b/ have short VOTs of around 0 msec, while voiceless sounds like /p/ have a longer VOT of around 60 msec and a boundary at around 20 msec. CP is thus observed when discrimination of two sounds with 40 and 60 msec VOTs (both /p/’s) is poor, but discrimination of 10 vs. 30 (a /b/ vs. /p/) is quite good. Because discrimination does not require overt labeling, this suggested that listeners can perceive acoustic differences that are relevant for discriminating categories but disregard differences that are not. CP suggests that listeners ignore any variability on this continuum, and one perceives a /b/ when it is below the 20 msec boundary no matter whether the VOT was 0, 10, or 15.

Theoretically, CP suggested that listeners solve the problem of lack of invariance by collapsing a continuous variable signal into discrete categories. That is, by ignoring within-category variation listeners could rapidly abstract a “quasi-symbolic” representation of the input (Goldstone and Hendrickson, 2010) such that a stimulus can be identified based solely on its relationship to the boundary: all VOTs <20 are /b/ and all VOTs greater than that are /p/.

**Figure 1:**
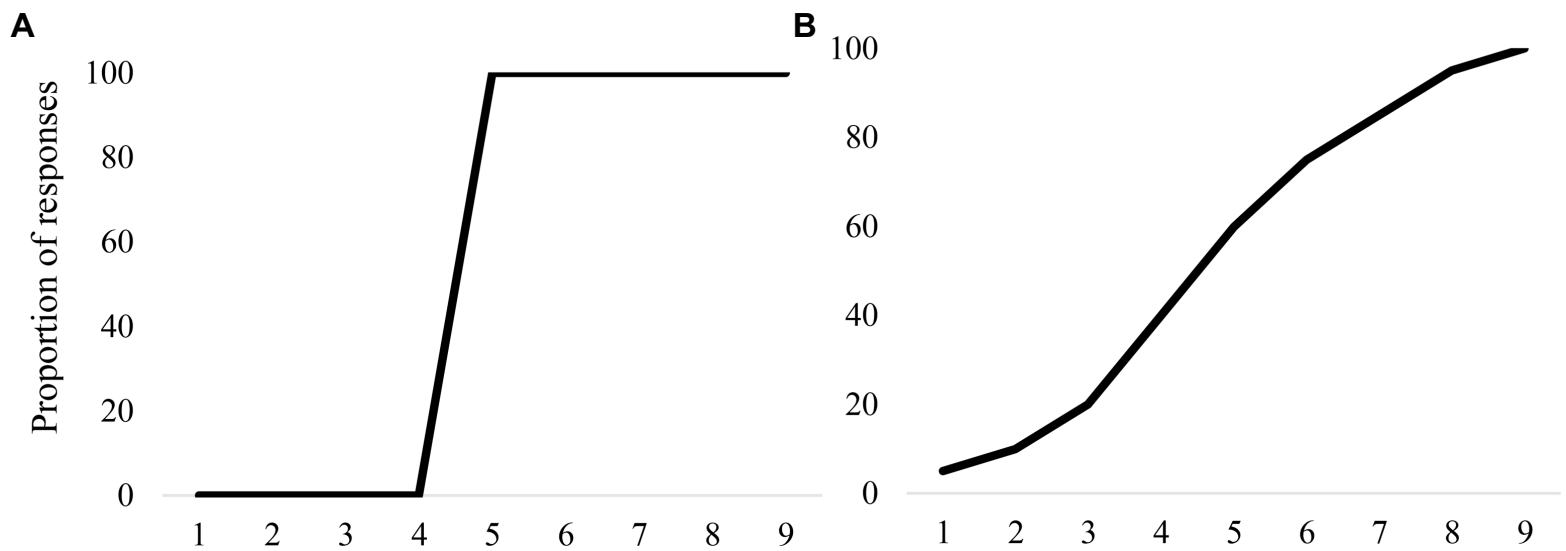
Proportion of responses in a typical 2AFC task where the black curve signifies the mean responses in both graphs. **(A)** A categorical profile which has a sharper slope. **(B)** A less categorical profile, with a shallower slope.

While discrimination tasks comprise the core empirical definition of CP, it is the forced choice identification task that has left the most vivid impact on fields like bilingualism. Empirically, forced-choice identification tasks require participants to listen to stimuli from a continuum and report which of several categories provided is the best match. If there are two, then the task is a two-alternative forced-choice task (2AFC), but larger response sets are possible (nAFC). What made this task so compelling is that in these tasks, monolingual or so-called typical listeners often show a near-perfect step function (Figure 1A), which seemed to capture the discrete nature of the system. Consequently, any deviation from this ideal may be informative.

Moreover, unlike discrimination tasks, nAFC tasks are feasible in diverse populations [i.e., younger children see (Slawinski and Fitzgerald, 1998; Hazan and Barrett, 2000); people with language impairments (Serniclaes, 2006); as well as bilinguals (Sebastián-Gallés and Bosch, 2002; Aoyama et al., 2004; Goriot et al., 2020)]. Many of these sorts of studies conducted on LX learners^1^ and clinical populations using forced-choice identification tasks use the slope of the categorization function as an index of speech categorization ability. Here, a categorical or step-like response function (i.e., sharper slope) is interpreted as having a “strong” ability (Serniclaes, 2006). Any deviation from being categorical is interpreted as a deficiency in the system (Figure 1B).

This interpretation aligns with the assumptions of CP as to how a good listener should behave – sharp categorical boundaries indicate that the listener ignores variation and successfully reaches the category decision. Having a shallow slope indicates some “deficit in categorical precision,” either through noise in the system or being unable to map the categories successfully (Serniclaes, 2006). When a bilingual individual does not show monolingual-like categorization in their second language or their heritage language, they are perceived to be differing from native-like proficiency levels (even as the concept of native-like proficiency is also arbitrary as not all bilinguals have the same goals or needs for proficiency). Indeed, the fact that both clinical language disorders and multilinguals show these kind of shallower response functions further emphasizes the deficit interpretation.

However, as we describe in the next section, mounting evidence shows that the interpretation of shallow slope reflecting a deficiency is problematic and does not capture the essence of speech categorization, even in normal hearing, “typical” monolingual listeners. If this is the case, differences in an nAFC task that are standardly interpreted from the lens of deficiency may in reality be driven from methodological and theoretical limitations and could reflect a unique approach to speech perception that may be more flexible or efficient for a bilingual. Therefore, before considering whether bilinguals’ have “native-like categories,” it is crucial to understand what categories are in monolinguals.

## Speech categories are gradient

Recent studies on speech perception have challenged the idea that speech input is carved into discrete categories and have shown clear evidence of gradiency in speech perception. While a history of studies has directly challenged the foundations of categorical perception using discrimination and other tasks to show continuous encoding (Massaro and Cohen, 1983; Schouten et al., 2003; Gerrits and Schouten, 2004; Toscano et al., 2010) our emphasis is not on criticisms of CP *per se*, but rather on a growing body of work that challenges the broader theoretical claim that discrete categorization is the ideal.

Evidence from studies that used priming (Andruski et al., 1994), continuous rating scales (Massaro and Cohen, 1983; Miller and Volaitis, 1989), the visual world paradigm with eye-tracking (VWP; McMurray et al., 2002, 2009; Kapnoula et al., 2021), and event-related potentials (ERPs; Toscano and McMurray, 2010; Sarrett et al., 2020; Kapnoula and McMurray, 2021) all converge on the idea that categorization is highly gradient. For instance, McMurray et al. (2002) tested monolinguals on a VOT continuum (e.g., spanning *beach* to *peach*) in a VWP task in which eye movements to each option were used to assess activation of the options (*beach* and *peach*) leading up to the ultimate nAFC response. They assessed *via* fixations to the picture of the competing word (e.g., *peach* when the target was *beach*), and found that this was linearly related to the continuous changes in VOT. That is listeners looked more to *peach* for a 10 msec VOT than a 0 msec VOT, even when they considered only trials where participants clicked on the target word (i.e., *beach*). This suggests that listeners are tracking continuous differences in VOT, within a category – not attempting to suppress these differences.

In fact, these gradient (rather than discrete) representations may be useful when listeners are coping with ambiguity and integrating different pieces of information in speech perception (McMurray et al., 2002, 2008, 2009; Clayards et al., 2008). For example, a more gradient representation may help listeners recover from misperceptions. McMurray et al. (2009) tested listeners on lexical garden paths words such as *ϸarricade*, where the onset sound came from a/b/to/p/continuum. Here, if the VOT was high (e.g., 40 msec), the word may be briefly interpreted as both *parakeet*, and resolution would not occur until late in the word (at-cade or-keet). However, if listeners were preserving gradient representations, they may be able to recover more quickly when the VOT was near the boundary. They found that if the VOT was around 40 msec, listeners were initially biased to interpret the input as the beginning of *parakeet* and then revised their decision when-cade arrives. In contrast, when the VOT was around 25 msec, listeners were still biased to *parakeet* but recovered faster because *barricade* was more active. If listeners were categorical, the activation of /p/should have fully suppressed the activation of/b/. In this case, a more gradient commitment (rather than a firm commitment to a discrete category) may help listeners be more flexible to integrate later cues efficiently to recover from misperceptions. Similar results have been seen with a variety of sources of ambiguity, suggesting that a partial commitment is the norm in speech perception (Szostak and Pitt, 2013; Brown-Schmidt and Toscano, 2017; Gwilliams et al., 2018).

Beyond flexibility, a gradient commitment is also important in learning and adaption, particularly when speech is inherently varied. Listeners need to learn and adapt to the talker’s speech to account for different factors such as their dialect, coarticulation patterns, rate of speech, or indexical differences. In fact, dozens of studies have documented the remarkable plasticity of speech perception (McQueen, 1996; Fenn et al., 2003; Bent et al., 2009). However, if listeners fully disregard fine-grained differences within a category, they would not be able to do this kind of learning (McMurray and Jongman, 2011). Importantly, these factors may interact. Clayards et al. (2008), for example, used a similar eye-tracking paradigm as McMurray et al. (2002), but with a perceptual learning twist. For some listeners, VOTs were highly consistent – most trials had VOTs near the prototypes for/b/and/p/with very little variation; for other listeners, VOTs were more variable. She found that after a brief exposure, listeners with high variance distributions adopted a more gradient representation. That is, people were learning to be more gradient when noise was expected (see also: (Theodore and Monto, 2019)).

This leads to the broader conclusion that underlying speech categorizations are highly gradient, and the degree of activation or consideration of one category or another is sensitive to fine-grained differences in continuous cues like VOT. However, it is unclear how to rectify this with traditional 2AFC tasks that show a sharp categorization. This is illustrated by a recent VWP study on the development of speech categorization. McMurray et al. (2018) used the same VWP paradigm from their 2002 study (McMurray et al., 2002) with children ages 7–18. Children heard tokens from VOT (e.g., *beach/peach*) or fricative spectra (*sip/ship*) continua and selected the corresponding picture while their eye movements were recorded as an index of lexical activation. The examination of the ultimate responses (i.e., the mouse click on the pictures) showed that older children had slightly steeper slopes. This appears to support the standard view – children’s categorization is getting steeper (more discrete) with development, and the younger children’s results mirror those of people with language impairments, or multilinguals (i.e., the association to the deficit model). However, the eye movements revealed a different story.

Similar to the McMurray et al. (2002) results, there was an overall gradient effect. As the VOT or fricative spectra moved toward the participant’s category boundary, there were increased looks to the competitor, indicating that children were sensitive to these fine-grained acoustic details. However, the youngest children showed the *least* sensitivity to these fine-grained acoustic details in the eye-tracking experiment, and this sensitivity grew with development. Under a quasi-discrete view, children with steeper slopes have *stronger* categories and should therefore be less sensitive to within-category detail. However, eye movements showed the exact opposite. In fact, it looks like children were achieving this sharper 2AFC categorization by becoming more sensitive to fine-grained details.

These findings have huge implications for how the slope of the identification is interpreted. However, beyond that, there are three deeper implications for work on multilingualism. First, even in monolingual children, speech perception skills develop slowly. This is unlike the most common views of speech categorization, which argues that speech categorization stabilizes in infancy (Werker and Curtin, 2005) (but see McMurray, 2022); critically in the context of multilingualism, it offers a gentle challenge to the notion that plasticity tapers off at later ages due to some kind of critical period – in fact, speech perception is developing quite slowly, implicating plasticity that may be available throughout the lifespan. Second, a gradient representation, rather than a discrete or categorical one, seems to be something desirable that people are attempting to develop. Finally, standard 2AFC tasks may show the complete opposite pattern of the underlying picture revealed by more sensitive measures like eye-tracking - a steep slope can accompany a highly gradient underlying representation.

This suggests serious problems with the traditional forced-choice identification tasks. In fact, it has long been known the discrimination tasks that formed the basis of support CP, involve other cognitive and decision processes that might create confounding factors for any given study (Gerrits and Schouten, 2004). However, nAFC identification is perhaps worse and the same pattern of data can be the product of completely different mechanisms of categorization (Kapnoula et al., 2017; Kapnoula and McMurray, 2021).

In classic two-alternative forced-choice tasks, listeners need to make a discrete judgment on a given trial. Because of the discrete nature of the response – and the effect of averaging – this can lead to enormous interpretative ambiguity. Consider a listener with a shallower-than-average identification curve (e.g., Figure 1B). Under the standard CP model, it would be assumed that this listener is responding variably from trial to trial—that is on some trials, a VOT of 15 msec (a /b/) is misperceived as a VOT of 25 msec (a /p/), leading to a different response. When averaged, we see a shallower curve. However, a shallow slope or a gradient profile might emerge from a completely gradient categorization. Here, listeners activate the competing category /p/ more near the boundary, and they attempt to approximate the frequency of their responses to the underlying gradient patterns. Therefore, they might respond 60% of the time indicating that the sound that they heard was a /d/ and 40% of the time that they heard /t/. These two profiles – both of which show shallower slopes - emerge from completely different underlying category structures. They cannot be differentiated from one another in a two-alternative forced-choice task. While the first scenario has a listener who does have discrete mapping between cues and categories, the second scenario has a listener whose underlying processes are gradient mapping.

The same is true for a steep (step-like) function. If we assume CP, this means that listeners have underlyingly discrete categories. However, if a gradient listener simply assumed a winner take all response mapping, one would see the same thing. That is, even if a speech token was perceived as 60% /b/like (e.g., near the boundary) if they always said/b/, one could observe a steep categorization curve even if the underlying categorization were gradient.

Thus, once we acknowledge that the underlying category structure could be gradient (as it clearly is in monolingual listeners), the 2AFC task is completely ambiguous. Clearly, not every shallower slope is due to gradiency – in many cases (e.g., hearing loss) it may be a marker of a problem. However, at the same time, in other cases, a shallower slope could be a sign of an adaptive and flexible gradient representation. Despite this ambiguity, both discrimination and forced-choice identification tasks are still widely used in language science research, and the assumptions that a steeper slope indicates more robust categorization are still commonly made. This is problematic not only for the larger language science community but also for research on bilingualism.

## Categorical perception and bilingualism

As we have described CP exerts a dominant force on the study of multilingual speech perception. In fact, the study of bilingualism has its own share of methodological and theoretical misconceptions (see for a review (Surrain and Luk, 2019)). Early bilingualism research was built on deficit models and in part due to methodologies adopted from other disciplines. Consequently, for a long time, bilingualism was treated as a discrete category in comparison to monolingualism, failing to consider variability in language experience, proficiency, and sociolinguistic contexts of each language (on the other hand see Bice and Kroll, 2019; Surrain and Luk, 2019; Bayram et al., 2021; López et al., 2021; Tiv et al., 2021; Castro et al., 2022; Kutlu et al., 2022). These early approaches specifically focused on LX learners’ ability to produce *native-like* utterances in their LX (Flege et al., 1995a; Piske et al., 2001; Alario et al., 2010). However, more recently, many scholars have begun to challenge this paradigm, assessing bilinguals on their own terms, given their own functional needs and environment. This work suggests bilingualism is better to be treated continuously and multi-dimensionally rather than as a discrete category (Surrain and Luk, 2019). In keeping with the classic views, the assumption of discrete categories and the methods of CP have also contributed to these deficit models. This shows up in at least two ways.

First, early bilingualism research assumed CP played a mechanistic role in explaining how well bilinguals learn categories or fully/partially transfer their L1 categories to their LX. That is, novel LX categories span regions of the perceptual space that lie within an L1 category (e.g., an English listener for whom the dental and alveolar/t’s/of Hindi lie within a single category). Given CP, people cannot hear these distinctions, causing a barrier in learning them.

The strength of this account led CP to become a dominant component of theories of bilingual sound acquisition (MacKain et al., 1981). This was in part due to the emphasis on perceptual narrowing in speech perception which argues that starting in the first year of life, infants lose the sensitivity to discriminate sounds in other languages but get better at discriminating contrasting sounds in the language that surrounds them (Best et al., 1988; Kuhl et al., 2006; Werker et al., 2012). Critically, this loss was seen as the end of a critical or sensitive period, blocking further plasticity (e.g., LX learning).

Second, bilingualism research also makes heavy use of the discrimination and identification tasks that were pioneered in monolingual speech categorization (Werker and Tees, 1987; Sebastián-Gallés and Bosch, 2002; Aoyama et al., 2004; Goriot et al., 2020). Given the assumption of CP, a shallower slope of the identification function has been associated with deficits or as an inability to map LX categories accurately due to factors such as age of acquisition or proficiency. Crucially, many studies linked early age of acquisition and higher proficiency to successful outcomes (i.e., steeper slopes) of discrimination tasks (Bosch, 2011). However, as we described, these identification tasks may not be truly estimating the nature of speech categorization. As we described in the previous section, a listener can have a steep curve while underlyingly having gradient categorization or they can have a gradient curve while having a steep curve underlyingly. This is the fundamental ambiguity of the slope function in a 2AFC task. It is unknown what the underlying mechanism is as the 2AFC task is not capturing these differences accurately.

What is interpreted as a shallow slope, and hence an inability to robustly categorize the stimulus, may actually be a mark of listeners’ flexibility and adaptation to categorizing overlapping categories. In fact, it may be almost impossible to impose fully discrete categories on the multiple phonological systems of a bilingual listener. Bilingual listeners need to adapt and learn from those cues more so than monolinguals. It is, therefore, more optimal to maintain a gradient mapping between cues and categories to permit more flexibility.

## Categorical perception in the context of heritage bilingualism

Most of the bilingualism enterprise in the late 90s through early 2010s primarily focused on balanced bilingualism (e.g., Peltola et al., 2012). This is the type of bilingualism where the use or the proficiency in both languages are somewhat equal. However, this picture of a bilingual as two monolinguals (Grosjean, 1989) does not accurately describe bilinguals who do not have the societal support or educational platforms to help them maintain their languages. For example, someone from a Spanish-speaking home in an English-majority country may only have access to a more specialized Spanish vocabulary (that which is needed at home) and may never learn to read Spanish, as their L2 (English) has much stronger support from school.

Historically, these minoritized bilinguals were consistently labeled as deficit language users (e.g., Bloomfield, 1927). That is, their abilities in their heritage language were seen as deficient (relative to a monolingual speaker of that language). To illustrate this point, consider two large bilingual populations in North America: (1) French/English bilinguals in Canada, and (2) Spanish/English bilinguals in Florida. If one strictly looks at age of acquisition for these two groups, it is possible to find early bilinguals in both contexts. It is also possible to find late learners of one of the languages in both contexts. What differentiates these two groups are primarily sociolinguistic factors.

In Canada, French is officially recognized as one of the two official languages spoken. Children in Canada (particularly Quebec) get some immersion in both languages, they are taught both in school, and they can maintain both Canadian French and Canadian English to some extent (even as bilingual groups in Canada who speak other languages and face other societal injustice toward their languages). Thus, many bilinguals in Canada are likely to fit the balanced bilingual definition, and many others are likely to be at least proficient in both languages.

In contrast, in the United States, cultural factors led to the stigmatization of Spanish (Kutlu and Kircher, 2021; Kircher and Kutlu, 2022), and as a result, immersion programs are rare and there are regional and racial disparities in access to general education in Spanish (Rosa, 2016). Children in the United States mostly do not receive any support in Spanish beyond the foreign language classroom, and for those who do, it is not sustainable at the national level. These children experience what is known as the heritage bilingual experience, where their home language is limited to the home settings due to societal prejudice and stigmatization. This prejudice is not only disrupting heritage speaker children’s heritage language development but also their bilingual development.

In these listeners, Spanish is perceived as a problem that needs to be fixed when bilingual children start schooling (Rosa, 2016). Children who have categories that are somewhat ambiguous in their comprehension and production are asked to fix this problem by immersing themselves in a solely English educational context (Rosa, 2016; García et al., 2021). The majority of the work on Spanish heritage-speaker children in the United States has argued that communities of minoritized languages should find ways to increase heritage-speaker children’s exposure to “native English speakers” to prevent them from having a gap in their English (Place and Hoff, 2011). Such recommendations use individual variability in development as a case for the assumption that heritage-speaker children cannot develop or are delayed in developing English proficiency as they are exposed to English at a later age or with a reduced amount while ignoring the social stigmatization towards bilingualism (Kutlu, 2020; Kutlu and Wiltshire, 2020).

Heritage speakers (of any language) are not a homogenous group (see Polinsky, 2018; Montrul and Polinsky, 2021). There are substantial differences in terms of exposure to the heritage language and the majority language, feelings of attachment to these languages, as well as perceived fluency in these languages. A survey of the literature reveals a wide variety of definitions and classifications of heritage bilinguals and heritage languages [e.g., (Benmamoun et al., 2013); also see (Ortega, 2020) for a detailed discussion]. In this context, there has been substantial work suggesting that heritage speakers are “deficient” in the majority language (Oller et al., 2007; Hoff, 2013), and that they cannot be considered “native speakers” of that language [but also see new conceptualizations on how heritage speakers can be placed in the native speaker continuum (Wiese et al., 2022)]. At the same time, research on heritage bilingualism has also focused on how heritage speakers retain and process their heritage language – which may also be deficient by this standard. This anti-nativization of heritage speakers from both their heritage and the majority language provided an array of places where heritage speakers experience a state of languagelessness (Rosa, 2016). Neither their heritage language nor their majority language fits into the “standard” norms. Much of the research on heritage bilingualism was done with the purpose of “fixing their languages” by providing more of the majority language.

Work on speech perception in heritage language speakers has the potential to fall into the trap of the deficit model. As we have described, the standard approach to speech perception in bilinguals was motivated by perceptual narrowing and CP (Caramazza et al., 1973; Werker et al., 1981; Werker and Tees, 1984; Flege, 1987; Flege et al., 1995b; Mayo et al., 1997; Sebastián-Gallés and Bosch, 2002, 2009; Bosch and Sebastián-Gallés, 2003; Aoyama et al., 2004; Kuhl et al., 2006; Garcia-Sierra et al., 2011; Stölten et al., 2014; Liu and Kager, 2015; Pan et al., 2022). In this context, any deviation in endpoints was interpreted as noisy encoding, deficiency in categories in their languages, or an unstable state of language use. However, a compelling alternative that cannot yet be ruled is that heritage speakers or bilinguals may be more gradient than monolinguals or individuals primarily exposed to one language or one language variety. This may serve to help them flexibly shift between languages.

Such interpretation does not require “fixing” a non-existent problem but focuses on the strengths of language-diverse individuals and how it informs our theories and methodologies. In fact, a theory based on deficiency arguments that do not consider language diversity has more potential to lead to educational outcomes that actually hinder language-diverse individuals from achieving the specific skills they need to navigate the educational system. That is an intervention designed to make such individuals perceive speech more categorically may actually be harmful. However, a fundamental limit is that the nAFC task simply cannot distinguish a noisier or poorer category representation from a more gradient one. Thus, to better inform theories of speech perception, we must move towards a continuous measure of speech perception.

## Moving away from categorical understanding of speech: Measuring gradiency

Given the interpretive ambiguity in the 2AFC task and the strong likelihood that categories are underlyingly gradient, there is a clear need for methods that can more directly assess this. Both eye-tracking and EEG studies have previously captured this and can clearly show the underlying gradient profiles (McMurray et al., 2002; Toscano and McMurray, 2010). However, using these methods is not trivial: they have large technical requirements, can be expensive, and have a great deal of trial-to-trial noise, requiring longer experiments.

In contrast, several recent studies have suggested that gradient categorization can also be measured by a simple behavioral experimental tool that can be implemented in online studies, lab studies, or field studies. This task, which we refer to as the Visual Analogue Scaling (VAS) task (Kong and Edwards, 2011, 2016; Kapnoula et al., 2017, 2021; Kapnoula and McMurray, 2021), is similar to the 2AFC task, however, as we argue below, its psychometric properties nearly eliminate the interpretive ambiguity of 2AFC.

**Figure 2:**
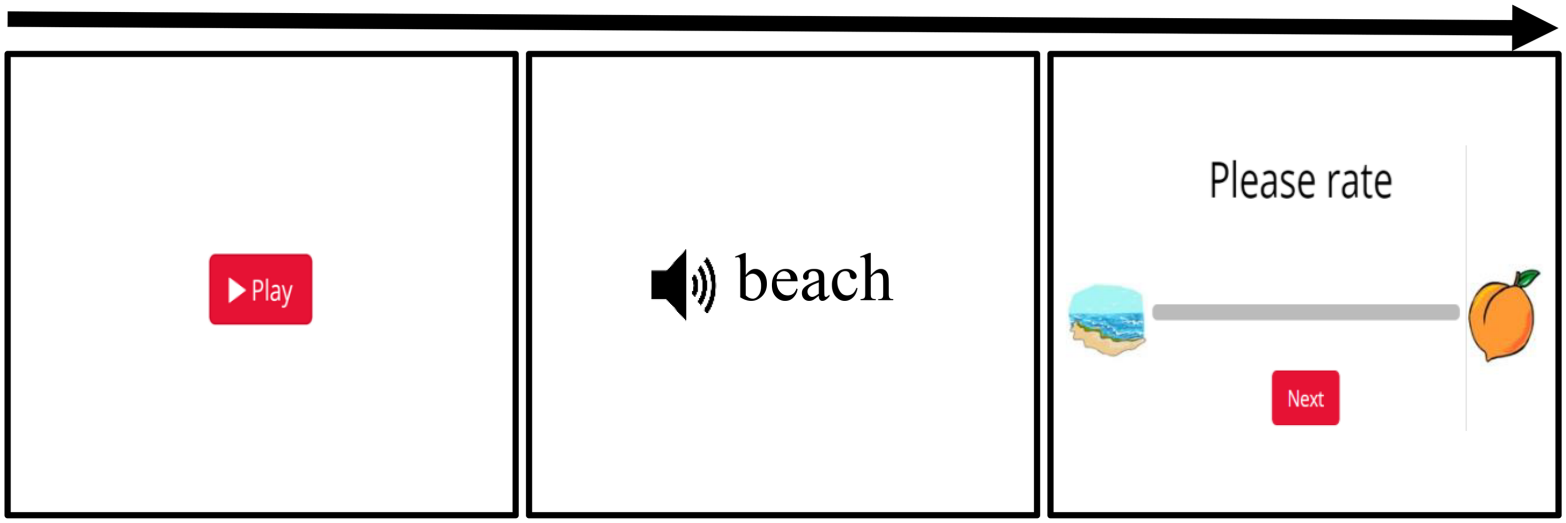
The layout of the experiment where the first panel shows the first page that the participants see when they are completing the online experiment. The second panel is when they hear the auditory stimuli. The last panel shows the VAS rating scene. Participants were asked to click on the scale to indicate where they think the auditory stimulus falls.

In the VAS task, as in the 2AFC task, a sound from a speech continuum is presented. However, instead of making a discrete binary choice, listeners are given a continuous scale on which to indicate where the sound falls between endpoints. For instance, if a listener is responding to members of a *beach*/*peach* continuum, the screen has an image of a *beach* on the left and a *peach* on the right with a straight scale in between (see Figure 2). They can then click anywhere on the line to indicate where they perceived this token.

In contrast to ERP and VWP tasks, the VAS task is straightforward and efficient to implement. It can be employed on any experiment building software (e.g., Experiment builder, PsychoPy, Matlab, Gorilla). It generally takes 15–20 min to complete with 2 to 6 repetitions per continua (moderate test–retest reliability (r = 0.48) of gradiency estimates was achieved with three repetitions of each stimulus see (Kong and Edwards, 2016)).

**Figure 3:**
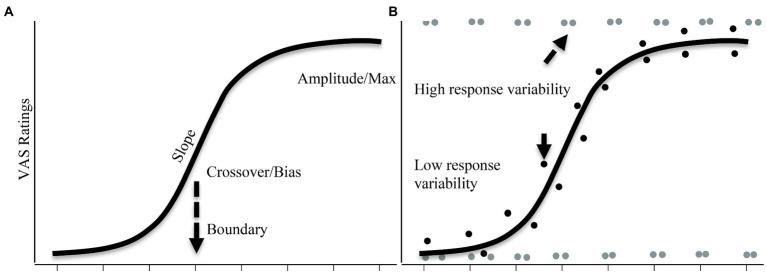
**(A)** Three parameters that can be extracted from VAS rating data. Averaged data across a continuum can provide measures such as amplitude, slope, and crossover/bias. **(B)** High response variability where a listener repeatedly uses the endpoints (in gray dots). This listener has underlyingly categorical representation. On the other hand, black dots show a listener who has an underlyingly gradient representation whose response variability is lower as their responses are tightly distributed around the average.

Critically, it overcomes concerns with the 2AFC task. Consider a situation in which listeners have a categorical or discrete boundary, but experience noise. In this case, the average VAS function should look highly gradient (like the 2AFC). However, if we look at individual trials, we should see that most trials have a VAS response that is near one endpoint or the other (Figure 3B grey points). That is, on each trial, they discretely heard /b/ or /p/, but shifts from trial to trial. In contrast, if the averaged response was because of an underlyingly gradient representation, we should see that individual responses are clustered tightly near the averaged (Figure 3B black points). Thus, by looking at individual trials relative to the average response, we can achieve more insight into the underlying nature of categorization.

Much of the recent work using the VAS task has examined individual differences in typical monolingual adults. For example, individual differences in gradiency in the VAS task predict the degree of gradiency in standard ERP and VWP paradigms (Kapnoula and McMurray, 2021), providing validation of the underlying constructs. It also predicts other skills. For example, adult listeners who more gradiently categorize stop voicing are more likely to use secondary cues (i.e., F0) when categorizing voicing (Kong and Edwards, 2016; Kapnoula et al., 2017), and they are better able to recover from misperceptions (Kapnoula et al., 2021).

Importantly, and most relevant to bilingualism research, in monolinguals there was little correlation between cognitive control tasks and the VAS task (Kapnoula and McMurray, 2021) or between gradiency in a non-linguistic visual continuum (Kapnoula et al., 2021) (e.g., an apple/pear visual continuum). This lack of an influence of more domain-general cognition may make it easier to isolate differences in speech perception in varying groups. Ongoing work in our lab is now successfully using this technique with monolingual children, monolingual children with bilingual exposure, bilingual adults, and cochlear implant users. Here we present preliminary data from a study in progress on bilingual adults to illustrate both how to use the VAS paradigm with this population, and how it can lead to greater clarity than prior approaches.

We note that this is an ongoing study and no statistical analyses have been conducted (as we have not reached our pre-planned sampling goal). Thus, our goal in presenting this data is not to make any specific claims about differences across bilinguals. Rather we examine this subsample of the data to illustrate how a more sophisticated approach to speech categorization could offer the kind of person-centered approach to speech that naturally fits with a more sensitive approach to bilingualism. Thus, our analyses are really meant more as a kind of case report for illustrative purposes, and we do not report many of the methodological details so as to avoid distracting from our goals here.

## A case study

To illustrate how we have used the VAS task (both methodologically and statistically) we present examples of data from an ongoing project. The goal of this project was to understand a diverse array of Spanish/English bilinguals in terms of relative proficiency, age of acquisition, social environment, and how these factors give rise to differences in speech categorization. We used online testing to recruit individuals with experience with Spanish and English in the United States and conducted an extensive language background questionnaire, social network questionnaire, and other measures along with a VAS task assessing eight different continua.

We note that the goal of this paper is solely to illustrate how we can assess gradient speech categorization in bilingualism research and separate it from poor categorization in a way that cannot be captured by standard nAFC tasks. Thus, we did not conduct any statistical analysis which awaits our true sample.

### Subjects

We have currently tested 73 listeners of various backgrounds. For ease of exposition below, we roughly group these subjects using age of acquisition to create four groups similar to those used in previous bilingualism research. The *Spanish-English heritage* (*n* = 32) speakers are defined as those with experience with both languages during the first 10 years of their lives and who also self-identify as dominant English speakers. The *L2 Spanish* (*n* = 9) group consists of individuals who acquired Spanish after the age of 10 only through schooling experiences, and for whom English is their dominant language. Next, participants in the *L2 English group* (*n* = 6) are those whose first language is Spanish and who acquired English as a second language after the age of 10. These individuals have the least dominance in English. Finally, the *English monolingual* group (*n* = 26) consists of speakers who acquired only English. These categories are designated solely for illustrative purposes and were not the groupings that originally motivated our ongoing study.

### Auditory stimuli

Auditory stimuli used in VAS experiments consist of monosyllabic minimal pairs in any language. Here, we provide an example of the eight continua used in our experiment. Our continua included two voicing contrasts (*beach-peach*, *dime-time*), five vowel contrasts (*beet-boot*, *bet-bat*, *pen-pan*, *hat-hot*, *net-nut*), and one fricative contrast (*sip-ship*). To construct the stimuli, we started by recording each endpoint word, spoken by an adult male with an American Mid-Western accent. The recordings were done in mono at a sampling rate of 44,100 Hz. Exemplars for endpoints were recorded in a carrier sentence to ensure uniform prosody and rate. We then selected one exemplar for each endpoint for each continuum.

VOT continua were created with a progressive cross-splicing procedure similar to (McMurray et al., 2008). Aspirated tokens were created by copying segments of the aspiration from *peach* and *time* and replacing the corresponding section of the onset of *beach* and *dime*, respectively. Fricative continua were created by a morphing procedure from McMurray and Jongman (2016). The frication portions from *sip* and *ship* were extracted, centered, and cut to be equal in length. Next, the spectral mean was calculated from the long-term average spectra. Both spectra were aligned to the average spectral mean. Then, weighted averages of the spectral shapes were extracted to create 0% /s/ to 100% /s/ in nine steps. Next, the frequency means of the spectra were shifted to create nine steps and a white noise filter was applied to each spectrum. Then, we imposed an average amplitude envelope on the filtered noise. Finally, the vowel continua were created by using TANDEM STRAIGHT (Kawahara et al., 1999). To create vowel continua, periodic information was first extracted for each endpoint. Then, temporal anchors were placed at the beginning, middle, and end of the target sounds. Spectral anchors were placed at the first and second formants. Finally, continua were morphed from one endpoint to the other across nine steps.

Visual stimuli were developed using a picture norming process adapted from McMurray et al. (2010). Candidates for stimuli were downloaded from a commercial clipart database^2^, then selected by a committee of undergraduate and graduate students for the most prototypical image. Images were then edited based on committee feedback (changing colors, removing or adding parts to the image), and edited to a uniform size and brightness.

### Procedures

The VAS task can be completed in the lab *via* touch-screen tablets and computers, or online *via* an internet browser. The example that is provided here was for online experiments which were implemented in Gorilla [^3^ (more about touch-screen testing with children can be found here: OSF^4^)].

In this task, participants hear a token from the continuum and report how closely it matched either endpoint by clicking along a line between the two pictures (see Figure 2). They are allowed to practice responding before proceeding to three practice trials, identical to experimental trials. After three practice trials, the participant begins the task.

On each trial, participants press a red PLAY button to initiate the word. After the word plays, the line appears and remains until the participant responds. Crucially, the line does not contain a slider or any marker until the participant makes a response (at which point a marker is shown). This avoids anchoring biases. The participant can change their response and indicate they are done by pressing the space bar.

Generally, when using multiple continua, we find it is much more efficient to present multiple trials from the same continuum in a block, and to maintain the sides of the pictures (e.g., the *beach* is consistently on the left and the *peach* on the right for some block of trials). This minimizes the amount of time that the participant needs to reorient to the task on each trial. However, in order to control for order effects and side bias, participants completed two blocks of each continuum which counterbalance the location of the endpoints along the response line. For example, the participant may see a picture of a beach on the left and a peach on the right in the first block, then see a *peach* on the left and a *beach* on the right in the eighth block (with blocks from other continua interspersed). Consequently, each continuum appears both early and late in the trial, with each endpoint on each side. In this study, each block consisted of 3 repetitions of 9 steps for each continuum or 27 trials/block. With two blocks for each of the 8 continua, this led to 432 total trials. The entire experiment took approximately 25–30 min.

## Parametric analyses of VAS

Typically, in a 2AFC task, listeners must judge the endpoints as 0 (e.g., /b/) and 1 (e.g., /p/). A classic step-function of CP is thus when tokens on one side of a category boundary would be marked as 0, and all tokens on the other side of the boundary would be 1. The VAS data are different in the sense that these data are on a continuous rating scale which reflects how close each stimulus is to the endpoints.

Previous work on VAS (Kong and Edwards, 2016) utilized a simple histogram to illustrate the way that listeners vary in their use of these continuous responses. This approach simply counts how often listeners respond to each point along the VAS. This method showed that some listeners used only the endpoints when responding, and others used the whole scale (i.e., more gradient).

However, Kapnoula et al. (2017) pointed out that this ignores the actual continuum step – a listener could have a flat histogram (a uniform distribution) because their responses perfectly match the continuum step (e.g., step 1 gets a low rating, step 2 gets a slightly higher one and so forth), or because they are just guessing. They thus introduced a parametric approach using non-linear curvefitting. They first computed the average response for each participant at each step. This was then fit to a nonlinear function. This function provides parameters like the slope of the function, the boundary, and the amplitude (the difference between the asymptotes; Figure 3A). These are described below and allow the researcher to directly characterize the shape of the function at an individual level.

To capture the nature of the trial-by-trial responses (e.g., Figure 3B), they then computed the difference between each individual trial rating and the mean. Therefore, if participants’ responses to individual trials overlapped with the mean, the sum of squared differences should be minimal, creating low response variability (true gradiency, Figure 3B, dark points). However, if the participants are choosing the endpoints, the individual responses should show a large deviation from the overall mean, leading to a higher sum of squared differences (high response variability, Figure 3B, light points).

The typical non-linear function is a four-parameter logistic function. These four parameters are (see Figure 3A): the slope, the amplitude (asymptotes), the crossover, and the bias. These parameters can efficiently capture gradient responses. When coupled with the response variation, these parameters can differentiate between a gradient pattern from a categorical pattern, and crucially a gradient pattern from a noisy pattern.

### Slope

The slope of the VAS dataset is analogous to that of a 2AFC task. It measures how the average function smoothly varies between the tokens or exhibits a more step-like function. However, as we have described in a 2AFC task, it is not possible to know whether a shallow slope emerges as a result of noisy or a more gradient encoding. The VAS task resolves this problem by incorporating response variability in the interpretation of the slope function as explained below.

### Amplitude

The amplitude indicates an overall difference between the asymptotes of the response function (i.e., position at which extreme end of the continuum). This measure often was ignored in 2AFC experiments, since the expectation was that in a forced-choice task the response should not be ambiguous (i.e., choosing one or the other category). However, in many groups, the endpoints of the continuum may never be unambiguous. For example, competition from an LX category could destabilize the response, or the particular acoustic cue that was manipulated in the continuum may not be the same cue the listener is expecting. The use of a logistic function with variable amplitude can eliminate this problem – particularly in a VAS task where listeners can respond to tokens in the endpoints continuously and are not required to use the ends of the scale. In fact, the differences in endpoint ranges may play a crucial role in understanding individual differences. Differences in amplitude parameter may be independent of differences in slope. For instance, a listener could have a low amplitude but steep slope or a low amplitude and shallow slope.

### Crossover

The crossover is the point where the function shifts from being in one category to the other (i.e., the category boundary). This is strongly analogous to the boundary seen in 2AFC tasks and can be used for similar inferences.

### Bias

The bias is the overall likelihood of a listener’s use of one end of the scale or the other end (e.g., the degree of vertical shift). This is introduced by the fact that the asymptotes need not reach 0 and 1.

**Figure 4:**
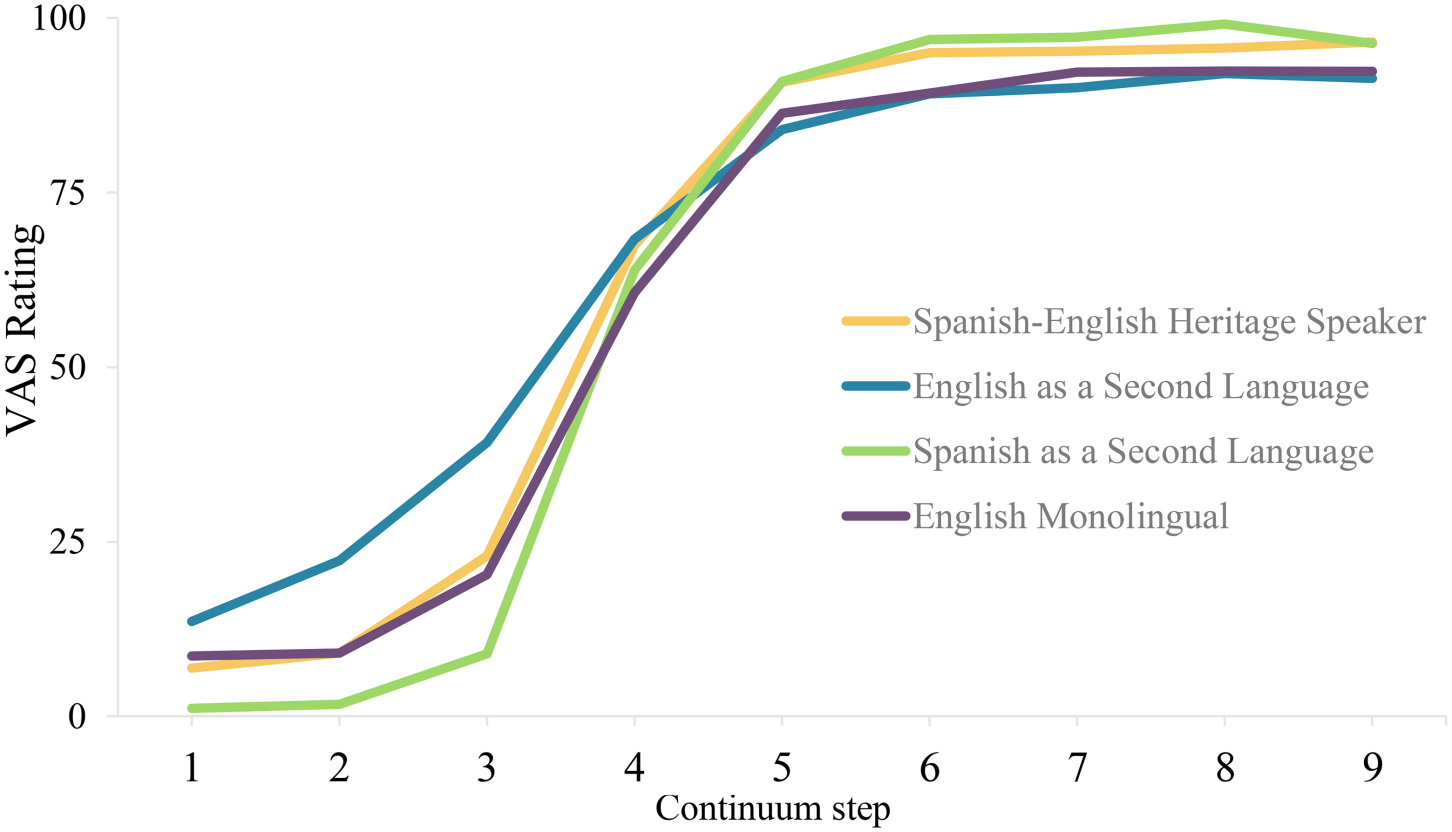
VAS ratings for four groups created based on Age of Acquisition of English and Spanish. Across four groups both Spanish as a second language and Spanish-English heritage speakers have the highest amplitudes. Spanish-English heritage speakers follow a more gradient slope compared to Spanish as a second language group.

To demonstrate, the average slope of each language group is shown in Figure 4. Here, we see that participants learning English as a second language (i.e., blue curve) has the lowest slope, followed by English monolingual speakers (dark purple) and Spanish-English Heritage speakers (gold), then followed by participants learning Spanish as a second language (green). Notably, there appear to be differences between Spanish-English heritage speakers and English monolingual speakers. The average amplitude of each language group in Figure 4 shows that participants learning Spanish as a second language and Spanish-English heritage bilinguals have the highest amplitude compared to the English monolinguals and English as a second language learners.

Moreover, participants learning Spanish as a second language seem to have a lower minimum and higher maximum response as compared to other language groups. English monolingual and heritage speaker groups seem to have identical minimum value. While these parameters visually present group differences, the VAS data provide further insight which is the calculation of response variability.

### Response variation

In addition to the parameters of the averaged estimated functions, we must also consider how closely the individual responses map onto these estimates. For example, a shallow average slope could arise from two distinct patterns of responding that cannot be captured by the 2AFC task but can be captured by the VAS task. First, a participant could be responding continuously to the different steps which results in a shallow slope with response points closely clustered around their slope. Alternatively, a shallow slope may emerge from a participant that responds primarily close to the endpoints of the line but does so inconsistently – the same step is categorized differently across responses. This latter pattern also results in a shallow slope when responses are averaged, but the majority of the data points would fall far from the average.

A close consideration of these patterns suggests that residual variation captures individual differences in three different profiles: categorical (steep slope), gradient (shallow slope + low response variability), and noisy but looks gradient (shallow slope + high response variability).

**Figure 5:**
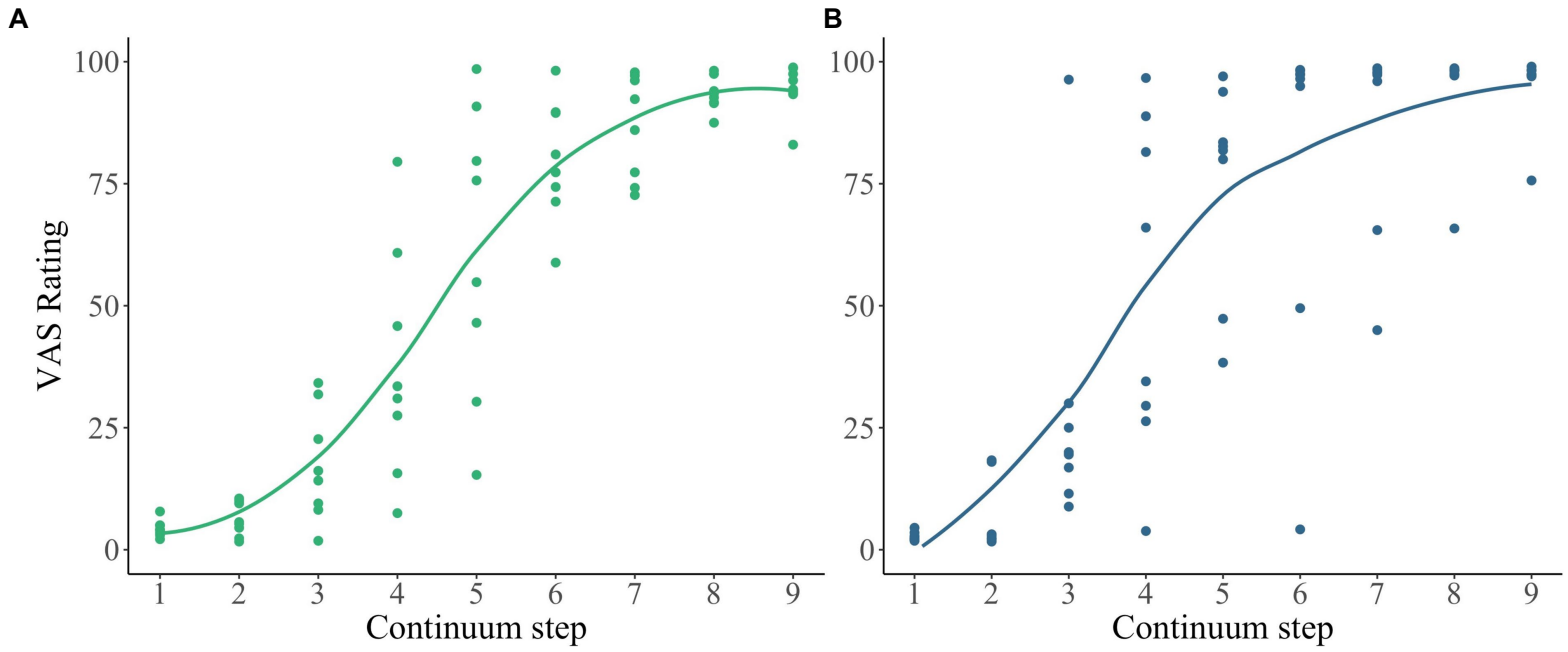
This figure shows the VAS ratings averaged across 9-steps for all continua for two participants. **(A)** Spanish-English heritage speaker who has a gradient profile with lower response variability. **(B)** English monolingual who has a categorical but somewhat noisy profile. Their response variability is larger compared to the heritage speaker.

**Figure 6:**
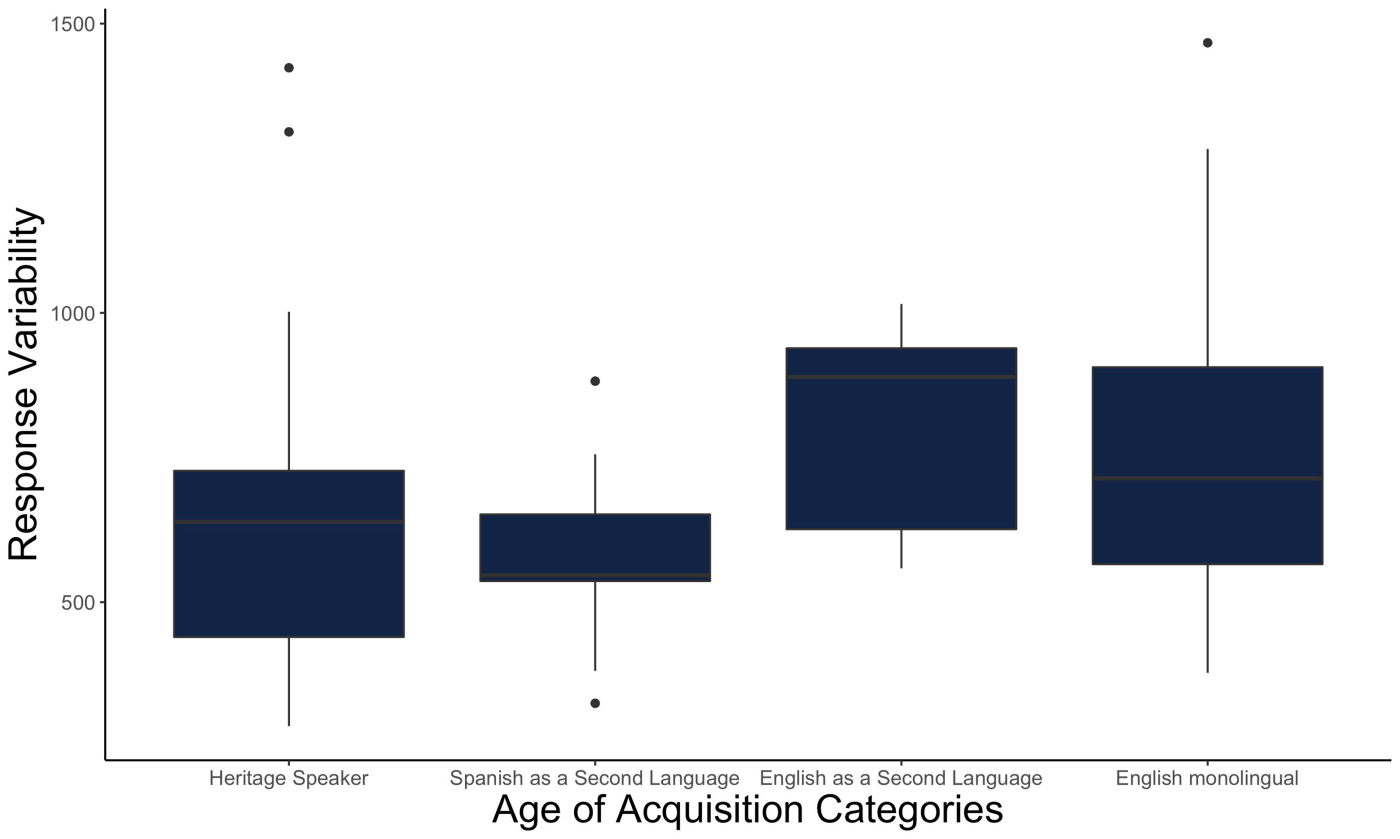
The residual variance calculated through the above formula on the y-axis. The higher the variance, the noisier the responses are. Here, we plotted four categories that were extracted from the Age of Acquisition variable from our dataset.

We use the parameters of the non-linear function to compute a response variability index. For this, we simply compute the predicted value for each step for that subject and then compute the mean squared difference of each individual point from the predicted value. If a participant is responding continuously, they will have low response variability and a shallow slope. On the other hand, a participant who inconsistently responds will have a large residual variation value and may have the same shallow slope. Fundamentally, both of these participants have shallow slopes. However, while one has a shallow slope because they integrate and use fine-grained details of the speech continuum, the other has it because of noisy encoding. Therefore, bilinguals showing shallow slope being interpreted as noisy encoding might in fact be the opposite of fine-grained gradient encoding (see Figure 5A). That is, by using slope (or amplitude) along with response variability we can differentiate a noisy response pattern from a more gradient or flexible categorization (Figure 5A versus Figure 5B). Figure 6 shows it clearly. Theoretically, we argue that response variability occurs when listeners disregard the acoustic cues in a noisy manner (i.e., being categorical but noisy). Here, we show that such noisy responses are also possible to see in English monolinguals.

We are currently developing new ways of analyzing VAS data that allow us to simultaneously estimate the logistic function and the response variability in the same multi-level model. In particular, as part of our co-registration for the *Growing Words* Project, we have proposed using a non-linear Bayesian mixed model (see more here^5^). However, this is still an ongoing effort to implement better statistical tools to investigate such data (see Figure 6).

## Limitations

As is the case with every experimental design, the VAS design also comes with its own limitations. For instance, it is unknown whether VAS has heavy demands on working memory. A listener who first categorizes the token and then converts their categorization to ratings might need to rely on their memory to remember the initial categorization. While this remains unknown, we argue that the VAS continuous responses are still a better option than an nAFC task as they minimally lack the interpretive ambiguity of that task. Future research should investigate whether bilingual and monolingual differences are driven due to potentially different memory use.

While the VAS could be combined with an analysis of reaction time, one challenge with this is that the use of RTs in the VAS task might not be ideal as listeners can change their decision before advancing to the next trial. If RTs are needed, we suggest that the experiment not allow the listeners the option to change their decision and provide instructions that encourage fast responding.

A third issue is that the VAS task is not the most ecological experimental setup for speech perception. It depends on computer-generated speech tokens which might not be possible to hear in the real world and also asks listeners to do something fairly unnatural with speech. However, at the same time, it may allow researchers to isolate processes involved in speech categorization that cannot be seen in other ways. Moreover, we point out that gradiency estimates from the VAS correlate with the much more natural VWP, which involves matching sounds to pictured referents (Kapnoula et al., 2021), suggesting this is not a major problem.

Finally, the development of analytic tools for the VAS is still an ongoing project. While different parameters can be extracted and frequentist statistics can be applied, Bayesian modeling might provide unique ways to delve into the rich VAS data. While these models are complex, open science practices that allow the sharing of scripts and data should allow researchers to practice their preferred way of approaching the VAS analysis.

## Conclusion

Classic work on bilingual speech perception has assumed categorical perception – both as a set of methods to be deployed and a theoretical “goal” of speech as providing quasi-discrete categories. In the context of bilingualism, the widespread use of these tasks suggested shallower identification slopes or poorer speech perception. This dovetailed with a deficit model of bilingualism in which any deviation from the monolingual performance was not accounted for until recently (Berthele, 2021; Wiese et al., 2022). Ultimately, this group of studies did not consider the fact that, just like with other language experiences, the bilingualism experience is not a static one. Bilinguals learn new languages, stop using those languages, or continue using those languages dominantly due to various personal and/or societal reasons (Kutlu and Kircher, 2021; Tiv et al., 2022). Considering language acquisition as a short period of the learning process that closes during the early years of childhood puts bilinguals such as heritage speakers into a never-ending gray zone. Heritage speakers were too bilingual for monolingual comparisons but too monolingual for bilingual comparisons. However, as research in monolinguals has abandoned both the method and the theory, this creates new opportunities for understanding bilinguals. In particular, speech categories are highly gradient and may be important for flexibility. This, along with a richer understanding of the diversity of the bilingual experience, demands new methods for understanding speech.

Thus, this paper described a new experimental method—the VAS task—that offers a more in-depth understanding of how bilinguals might categorize speech sounds. This may help avoid a deficiency argument by allowing the researcher to better characterize the process along multiple dimensions, by helping to identify structural gradiency in categorization (which may be adaptive), and to discriminate it from patterns that reflect difficulty. Language science research is moving more towards such gradient analysis in other fields as well (Levshina et al., 2021), and our own contribution here builds on this important trend. Importantly, methods that embrace this kind of gradiency may ultimately help build a more interdisciplinary approach to language science as not all subfields of language sciences have historically ignored variation (i.e., years of sociolinguistic research that examine variation). Moreover, our argument is consistent with broader trends in psycholinguistic research to continuously integrate an understanding of variability (both within and across individuals) in our methods and theories (Titone and Tiv, 2022).

Forced-choice tasks may be useful in some contexts including work on bilingualism. However, if the primary concern is the slope or steepness of the function, this task is highly ambiguous, and a shallow slope could be due to a deficiency or to increased gradiency. Consequently, this task may lead to the interpretation of monolingual-bilingual differences as deficiencies, which may in fact not reflect the reality of speech perception neither in monolinguals nor in bilinguals. In fact, for listeners who are surrounded by variability in their everyday lives, gradiency might be more ecological and cognitively efficient than a discrete representation. Furthermore, bilingual research continues to move away from trying to fix so-called deficiencies that do not exist (Bayram et al., 2021). The VAS task, along with many recent theories and methodologies, is one of the tools that can continue to provide researchers with tools to account for individual differences.

## Data availability statement

The original contributions presented in the study are included in the article/supplementary material, further inquiries can be directed to the corresponding author.

## Author contributions

EK and BM contributed to conception and design of the study. SC organized the experiment. EK and SC performed the analysis. EK wrote the first draft of the manuscript. BM and SC both contributed to the writing and editing of this manuscript. All authors contributed to manuscript revision, read, and approved the submitted version.

## Funding

This work was supported by NSF grant number 2104015 to EK and BM, and NIH grant DC 008089 to BM.

## Conflict of interest

The authors declare that the research was conducted in the absence of any commercial or financial relationships that could be construed as a potential conflict of interest.

## Publisher’s note

All claims expressed in this article are solely those of the authors and do not necessarily represent those of their affiliated organizations, or those of the publisher, the editors and the reviewers. Any product that may be evaluated in this article, or claim that may be made by its manufacturer, is not guaranteed or endorsed by the publisher.

## Footnotes

^1^We use the term "LX learners" to embrace the diversity of L2, L3, heritage language speakers and other forms of multilingualism (Dewaele, 2018).

^2^ https://clipart.com

^3^ www.gorilla.sc

^4^ https://osf.io/q39yt

^5^ https://osf.io/q39yt
